# Preslaughter factors affecting mobility, blood parameters, bruising, and muscle pH of finished beef cattle in the United States

**DOI:** 10.1093/tas/txae035

**Published:** 2024-03-13

**Authors:** Paxton A Sullivan, Melissa K Davis, Mahesh N Nair, Ann M Hess, Daniel F Mooney, Lily N Edwards-Callaway

**Affiliations:** Department of Animal Sciences, Colorado State University, Fort Collins, CO 80523, USA; Department of Animal Sciences, Colorado State University, Fort Collins, CO 80523, USA; Department of Animal Sciences, Colorado State University, Fort Collins, CO 80523, USA; Department of Statistics, Colorado State University, Fort Collins, CO 80523, USA; Department of Agricultural and Resource Economics, Colorado State University, Fort Collins, CO 80523, USA; Department of Animal Sciences, Colorado State University, Fort Collins, CO 80523, USA

**Keywords:** Cattle welfare indicators, dark cutting, fed cattle, meat quality, preslaughter stress, truck waiting time

## Abstract

Decades of work have focused on reducing fear, stress, and discomfort in cattle during the preslaughter phase by improving and promoting animal handling, transportation, and management processes. Even still, there is limited information about the effects of preslaughter factors on animal welfare and meat quality outcomes in finished cattle in the United States. This study aimed to track individual animals through the slaughter process to identify preslaughter factors associated with key welfare and quality outcomes. A total of 454 cattle from one commercial slaughter facility were studied. Preslaughter factors assessed included distance traveled, truck waiting time, lairage density, lairage duration, and season. Animal characteristics, i.e., body weight, breed, and sex, were also recorded. One trained observer scored the mobility of all cattle using the North American Meat Institute’s 1-4 scale (i.e., normal to extremely reluctant to move). Exsanguination blood was collected and analyzed for cortisol, creatine kinase, and lactate. Carcass bruising was scored using a modified version of the National Beef Quality Audit’s bruise-scoring methodology (i.e., no bruise, one bruise ≤ the size of a deck of cards, one bruise > than the size of a deck of cards, and multiple bruises). Ultimate muscle pH was measured 32 to 36 h postmortem. Multi-predictor models were selected for each outcome variable using Akaike Information Criterion. Continuous outcome variables were analyzed using linear mixed-effect models and categorical outcome variables with mixed-effect logistic regression models. Longer truck waiting times were associated with increased cortisol (*P *= 0.04) and lactate (*P* = 0.02) concentrations. Similarly, increased lairage duration was associated with increased creatine kinase concentrations (*P* = 0.05) and the odds of cattle being bruised (*P* = 0.03). Less space allowance per animal in lairage was associated with increased odds of cattle having impaired mobility (*P* = 0.01). There was a seasonal effect for many of the measured outcomes; the summer season was associated with greater lactate concentrations (*P* < 0.0001), increased odds of impaired mobility (*P* < 0.0001), and increased odds of carcass bruising (*P* = 0.003). The findings of this study indicate that many of the preslaughter factors assessed influence critical welfare and meat quality outcomes of finished beef cattle, warranting future research and consideration.

## Teaser

This research investigates the complex relationships between preslaughter factors, animal welfare, and meat quality outcomes in a population of finished beef cattle. Several factors, including truck waiting time, lairage density and duration, and season were associated with physiological stress parameters, cattle mobility, and carcass bruising, further highlighting the importance of the preslaughter period for animal welfare.

## Introduction

Heightened consumer awareness and interest in the welfare of food animals has not only influenced care and use standards for food animals across various sectors (Broom, 2010; Clark et al., 2016; Alonso et al., 2020) but has also prompted corporations to integrate animal welfare as a central component of their business ethos and corporate social responsibility initiatives (Maloni and Brown, 2006; Sullivan et al., 2017). While policies and practices aimed at safeguarding and promoting animal welfare during preslaughter processes have been long established as a critical component of beef cattle slaughter (HMSA, 1978; Grandin, 2006; Grandin, 2012), undoubtedly, cattle may still undergo varying levels of stress as they are marketed through the final stages of the beef supply chain (Chulayo et al., 2012; Edwards-Callaway and Calvo-Lorenzo, 2020). This final marketing phase encompasses numerous inherent stressors introduced at different time points, from the loading of cattle onto trucks at feedlots to their transportation to processing plants and, finally, their resting period in lairage pens before slaughter.

In response to stressful stimuli, cattle may elicit a range of physiological reactions that are measurable and serve as helpful tools for assessing animal welfare at slaughter (Grandin, 2014). Creatine kinase (**CK**), lactate, and cortisol are examples of physiological stress indicators routinely measured to assess the stress response of cattle to events such as handling and transport (Losada-Espinosa et al., 2018). CK and lactate are especially useful when assessing stress associated with physical effort or fatigue (Gruber et al., 2010; Thomson et al., 2015; Hagenmaier et al., 2017), while cortisol—one of the most widely used indicators of stress in cattle—is used to determine an animal’s response to both acute and chronic stressors (Tadich et al., 2005; Romero et al., 2017; Abubakar et al., 2021). However, cortisol levels can be highly variable and not always indicative of adverse events, so it is typically analyzed alongside other parameters. Postmortem, meat quality defects can also be measured to assess the extent to which an animal has coped with stress in the preslaughter period (Sullivan et al., 2022). Chronic stress prior to slaughter can initiate the antemortem catabolism of muscle glycogen, which, if significant, can inhibit the normal postmortem pH decline of the muscle, resulting in beef that is too high in pH with a characteristic dark, purplish-red color (Ponnampalam et al., 2017). This quality defect, known as dark-cutting beef, has a shortened shelf-life (Newton and Gill, 1981) and is visually unappealing to consumers (Jeremiah et al., 1972). While the suite of physiological welfare indicators is vast, collecting and maintaining the integrity of blood samples at the time of slaughter can require substantial preparation, labor, and data collection inputs, particularly in large commercial facilities.

Other less expensive and noninvasive indicators of cattle welfare, such as cattle mobility and carcass bruising, have been developed and utilized to observe the impact of preslaughter factors on animal welfare (NAMI, 2016; Texas A & M University, 2016); these indicators are included in industry benchmarking efforts (e.g., Elanco’s Cattle Mobility Assessment Database and the National Beef Quality Audit (**NBQA**), Eastwood et al., 2017) and used to evaluate and aid in the continual improvement of cattle welfare. Even though most cattle arrive at plants with normal mobility (i.e., 96.8% with a score of 1, Eastwood et al., 2017), impaired mobility still poses significant welfare challenges, as there is some level of pain associated with lameness (Shearer et el., 2013; Whay and Shearer, 2017). Carcass bruising, attributed to factors like rough handling (Mendonça et al., 2018) and high stocking densities (Ferreira et al., 2020), impacts both welfare and economic value (Strappinni et al., 2010; Huertas et al., 2015; Lee et al., 2017) and therefore warrants significant attention to reduce its prevalence.

While some preslaughter factors like transportation (Tarrant, 1990; Knowles, 1999; Schwartzkopf-Genswein et al., 2012), animal handling (Warriss, 1990; Grandin, 2014; Gallo et al., 2022), and lairage practices (Gallo et al., 2003; Liotta et al., 2007; Teke et al., 2014) are well-researched and their relationships to animal welfare well understood, other factors such as truck waiting times to unload at the plant, lairage pen density, and the use and efficacy of heat mitigation techniques in lairage are underrepresented in the current body of work (Davis et al., 2022a; Sullivan et al., 2022). It is also challenging to draw conclusions from studies conducted in varying parts of the world and with diverse cattle populations, as the final marketing phase varies considerably across the globe, for example, in transport type and conditions, lairage duration, and slaughter practices. To elucidate the complex relationship between preslaughter factors and animal welfare further, a recent large-scale study was conducted to benchmark current preslaughter practices, cattle welfare, and meat quality outcomes in a population of approximately 80,000 finished beef cattle in the United States (Davis et al., 2024). However, most of the available information is observed or measured in groups of animals (i.e., slaughter lots) and not on individual animals. Therefore, the objective of the present study was to track individual animals throughout the slaughter process to identify preslaughter factors associated with key welfare and quality outcomes in finished beef cattle in the United States.

## Materials and Methods

### Ethical Statement

Due to the noninvasive nature of all animal measurements and observations taken as a part of this study, an exemption was filed and granted by the Colorado State University Animal Care and Use Committee (IACUC Exemption #2019-080-ANSCI).

### Slaughter Facility and Cattle Population

For 1 wk each in January and July 2022, data were collected at one federally inspected slaughter plant in the Southwestern region of the United States (US) that processes fed cattle. The slaughter facility operates two 8-h production shifts, with an average slaughter capacity of 5,000 cattle per day and a chain speed ranging from 325 to 375 cattle per hour. Data were recorded over two periods to capture variability in weather across the winter and summer seasons (represented by January and July, respectively; Table 1). Due to logistical and personnel capabilities and the complexity associated with tracking animals throughout the slaughter process, samples were taken only from cattle slaughtered on the first shift, which ran from approximately 0630 to 1500 hours each day. Researchers tracked individually marked animals throughout the slaughter process and collected both antemortem and postmortem measurements.

**Table 1.** Minimum, mean, and maximum ambient temperature, humidity, and THI by season

Season	Temperature^1^ (°C)			Humidity^1^ (%)			THI^2^, Index		
	Min	Mean	Max	Min	Mean	Max	Min	Mean	Max
Summer^3^	26.70	33.90	42.20	13.00	25.20	43.00	73.00	78.20	83.80
Winter^4^	−6.11	8.33	15.60	14.00	32.20	68.00	27.60	50.20	59.00

^1^Temperature and humidity were recorded daily using an online commercial weather service (Weather Underground, San Francisco, CA, USA).

^2^THI was estimated using the following equation: THI = 0.8*T + RH*(*T* − 14.4) + 46.4, where *T* denotes temperature in °C and RH denotes relative humidity as a proportion (LiveCorp and Meat and Livestock Australia Veterinary Handbook, 2023).

^3^The summer season includes data collected in July 2022.

^4^The winter season includes data collected in January 2022.

All cattle in the study were handled according to the plant’s standard operating procedures, including being unloaded from a truck, moved into lairage pens by facility employees, and inspected by a United States Department of Agriculture (**USDA**) veterinarian before slaughter. During antemortem inspection, cattle were moved out of their pen to another pen of a comparable size. In the lairage pens, cattle were provided with ad libitum access to water and, in the summer season, were cooled with sprinklers when temperatures exceeded 26.7 °C. All lairage pens and handling areas had stamped concrete flooring and were uncovered. After being inspected, cattle were moved through the handling area to a center-track restrainer, where they were rendered insensible with a pneumatic captive-bolt stunner (Jarvis Products Corporation, Middletown, CT, USA) and subsequently exsanguinated before further processing.

A total of 454 cattle were included in this study (Winter, *n* = 199; Summer, *n* = 255) which originated from commercial feedlots. Individual live weights were not measured at the slaughter facility; consequently, live weights for individual animals were estimated by dividing the animal’s hot carcass weight (**HCW**) by the dressing percentage (**DP**) for the animal’s respective slaughter lot.

### Individual Animal Identification

Trucks arrived from approximately 0400 to 1130 hours daily for the first shift. Every truck’s arrival time, defined as the time a truck arrived at the plant regardless of if they had to wait outside a fenced-in area before checking in, was recorded. Unloading time was recorded at a single timepoint and was defined as the time the first animal walked off of the truck and onto the unloading dock. The time cattle spent waiting on the truck at the plant (i.e., truck waiting time) was calculated by subtracting a truck’s time of arrival from the time cattle were first unloaded from it. The first and one of the last two trucks to arrive from a slaughter lot were chosen for study inclusion to capture variation in truck waiting times. Approximately ten slaughter lots (i.e., twenty trucks) were sampled per day.

From each study truck, five to six animals were selected and marked during unloading to track them throughout the entire slaughter process; the selection process aimed to maintain as much randomness as possible, although it was constrained by the researchers’ proximity to the animals being marked and their ability to safely place the marking. Consequently, cattle positioned inside the group of animals on the pen scale were not marked. Animal-related factors such as sex, hide color, and size (including height or weight) were not considered by the researcher during the selection process. One deck of cattle was unloaded at a time (approximately 20 animals) and were moved into a pen scale, where two researchers individually marked cattle with a livestock breeding patch (Estrotect Heat Detector; Rockway Inc., Spring Valley, WI, USA) and applied with adhesive glue (Kamar Adhesive; Kamar Products Inc., Zionsville, IN, USA). The patch was placed along the dorsal topline of each animal, usually between the shoulders or on the rump. Multiple patch colors were used to distinguish individual animals within a slaughter lot.

Google Maps (Google LLC, Mountain View, CA, USA) was used to calculate an estimated transport distance between the feedlot of origin and the plant. When multiple routes were available, the first route suggested by Google Maps was selected; this route typically represented the route with the shortest distance. It should be noted that the variable “distance traveled” is an estimate and does not necessarily reflect the exact distance cattle were transported prior to arrival to the plant.

### Mobility Scoring and Open-Mouth Breathing

Cattle mobility was scored at a single timepoint using the North American Meat Institute (**NAMI**) mobility scoring system (1 to 4 scale; normal to extremely reluctant to move; Table 2; NAMI 2016). One researcher with extensive experience assessing fed cattle mobility scored all cattle included in the study from a catwalk above the lairage pens, typically when the animals were being moved from their holding pen to the staging area just before slaughter. A second researcher recorded an individual animal’s hide color (i.e., black hided, not black hided) and breed type (i.e., predominately *Bos taurus* vs. predominantly *Bos indicus*). Cattle were determined to have *Bos indicus* influence when they possessed two or more typical breed characteristics, including having a large hump above the shoulders, droopy ears, and excess skin on their dewlap and prepuce; only 3% of all cattle sampled met this criterion. In addition to mobility, open-mouth breathing was assessed in the lairage pens using a modified version of the panting scoring system developed by Mader et al. (2006). However, due to the absence of signs of heat stress observed by the researcher, heat stress was not analyzed in the present study.

**Table 2.** Mobility scoring system for finished cattle developed by the NAMI

Mobility score	Definition
1	Normal, walks easily, no apparent lameness, no change in gait
2	Exhibits minor stiffness, shortness of stride, slight limp, keeps up with normal cattle
3	Exhibits obvious stiffness, difficulty taking steps, obvious limp, obvious discomfort, lags behind normal cattle
4	Extremely reluctant to move even when encouraged, statue-like

### Blood Collection and Analysis

Exsanguination blood was collected from all study animals. Immediately after exsanguination, researchers collected blood in a plastic cup and subsequently transferred it into two prelabeled tubes: a five mL glucose determination tube with additives of Sodium Fluoride as a stabilizer and Potassium Oxalate as an anticoagulant, and a ten mL serum tube with silicone coating as a clot activator (BD Vacutainer; Becton, Dickson and Company, Franklin Lakes, NJ, USA).

Approximately 30 min after collection, whole blood was analyzed for lactate with a handheld lactate meter and test strip (Lactate Plus; Nova Biomedical Corp., Waltham, MA, USA). The remaining blood tubes were left to clot at room temperature for approximately 30 min before being centrifuged (Centrifuge 5810; Eppendorf North America, Framingham, MA, USA) at 2,000 × g for 15 min at 4 °C. The resulting serum was pipetted into two microcentrifuge tubes and placed on dry ice for transport; samples were stored at − 80 °C until further analysis by a third-party laboratory. The serum was analyzed for CK using a Cobas C 501 Analyzer (Roche Diagnostics, Indianapolis, IN, USA). Additionally, serum cortisol was assayed in duplicate using a commercial radioimmunoassay kit (ImmunoChem Cortisol Coated Tube RIA kit; MP Biomedicals, LLC., Santa Ana, CA, USA). Project intra- and interassay coefficients of variability for serum cortisol averaged 12.8% and 13.3%, respectively.

### Meat Quality

#### Bruise scoring

After the hide was removed but before the carcasses were split, three trained researchers (in rotation) scored carcass bruising and recorded carcass identification numbers. Researchers were trained against a bruise-scoring expert using a series of in-plant videos and real-time experience and had to earn a Kappa coefficient, an estimate of intra-observer reliability, of 0.80 or higher to qualify for bruise-scoring. The methodology employed in this study was adapted from the NBQA bruise-scoring system, which uses a 10-point scale to estimate bruise size and weight visually. The scale employed in the present study used four categories based on the upper bound of the NBQA’s “Minimal” category (i.e., a bruise the size of a deck of cards) to simplify bruise assessment in real-time at fast chain speeds. The four categories were no bruise, one bruise that was ≤ the size of a deck of cards, one bruise that was > than the size of a deck of cards, and multiple bruises (the size was noted); all categories were mutually exclusive.

#### Carcass characteristics

Carcass processing occurred according to the plant’s standard operating procedures. After carcasses were chilled at −2 to 1 °C for approximately 24-h postmortem, plant employees ribbed carcasses between the twelfth and thirteenth ribs before USDA employees evaluated and ranked carcasses for USDA quality and yield grades. In addition to USDA grades, HCW, DP, and dark cutter data were obtained for each carcass from plant records.

#### Muscle pH

Approximately 32 to 36 h postmortem, muscle pH was determined by using a portable pH meter. Measurements were determined with the Hanna Portable Meat pH Meter (Hanna Instruments, Woonsocket, RI, USA) for the first data collection, but due to in-plant environmental conditions, the Orion Star A121 Portable pH Meter (Thermo Scientific, Waltham, MA, USA) with the Orion 8104BN ROSS Combination Rugged Bulb pH Electrode (Thermo Scientific, Waltham, MA, USA) was used for the second data collection. In addition to the electrode, the Orion Stainless-Steel Automatic Temperature Probe (Thermo Scientific, Waltham, MA, USA) was used to compensate for variations in pH measurements due to changes in ambient temperature. The probe was inserted into the exposed longissimus thoracis muscle (referred to as the ribeye) between the twelfth and thirteenth ribs on the right side of the carcass. A single measurement was taken for each carcass. The probe was rinsed with de-ionized water and blotted dry between measurements, and instrument calibration was conducted every 20 samples.

### Statistical Analysis

All statistical analyses were performed using R Statistical Software (v4.2.2; R Core Team, 2021), with individual animals serving as the observational unit. Descriptive statistics were calculated for all variables of interest; continuous variables were summarized by their minimum, mean, maximum, and standard deviation (**SD**), and categorical variables by their relative frequency. For analysis purposes only, some variables were collapsed into smaller categories. Due to the low frequency of mobility scores 3 and 4 observed, mobility was categorized as a binary variable (normal mobility, i.e., a score of one; impaired mobility, i.e., a score of 2 or higher). Bruise scores were similarly collapsed into a binary variable for analysis (none/minimal, i.e., no bruise or one bruise ≤ the size of a deck of cards; greater than minimal, i.e., one bruise > than the size of a deck of cards or multiple bruises of any size).

#### Regression analysis

Mixed model analysis was used to account for lot effects as there were multiple animals observed per slaughter lot. Continuous variables, including cortisol, CK, lactate, and muscle pH, were analyzed using linear mixed-effect models (lme4 v1.1-31 and lmerTest v3.1-3); diagnostic plots were used to assess model fit and ANOVA assumptions. To account for nonnormality, a logarithmic transformation was performed on CK data for analysis and later back-transformed for reporting purposes. Mobility was analyzed using a mixed-effect binary logistic regression. Bruising was analyzed using a binary logistic regression; in this model, high model complexity generated a nonconvergence issue. To solve this issue, slaughter lot was excluded as a random effect in the bruising model only.

Type III F-tests (car v3.1-1) were used to analyze all models. Additionally, pairwise comparisons for categorical variables were calculated using estimated marginal means (emmeans v1.8.4-1). Statistical significance was determined at *P* ≤ 0.05 and statistical trends were declared at 0.05 < *P* ≤ 0.10. The following results are reported as (*n*, %) unless otherwise noted.

#### Model selection

Akaike Information Criterion (**AIC**) was used to evaluate regression models with multiple predictors. The variables season, body weight, sex, breed, truck waiting time, distance traveled, lairage density, lairage duration, mobility (except for the mobility model), and bruising (except for the bruising model) were considered for all full models during the model selection process. In addition, lactate was added as a predictor variable for the mobility and pH models. For each response variable of interest, a full model was fit separately (meaning all possible and relevant predictor variables were included), an automated model selection was applied (package: MuMIn v1.47.1), and the model with the lowest AIC was selected. For a subset of response variables, a small number of two-way interactions were included in the model selection process based on their relevance to the specific research question and previous subject matter knowledge (e.g., season by lairage duration); however, none of the lowest AIC models included interactions and were therefore excluded from further analysis.

## Results

Population characteristics are presented in Table 3. In brief, the individually identified cattle population included fed steers (287, 63.22%) and heifers (167, 36.78%) from local commercial feed yards that were predominately black-hided with *Bos taurus* influence (286, 63.70% and 438, 96.90%, respectively). The study population had an average live weight of 610.67 ± 71.61 kg (mean ± SD) and ranged from 371.38 to 837.07 kg. Postmortem, study animal HCWs ranged from 237.68 to 530.70 kg with a mean of 388.43 ± 45.45 kg. In addition, the average DP in this study was 63.60 ± 0.70% and ranged from 61.80 to 65.10%.

**Table 3.** Animal characteristics of study cattle, including sex class, breed type, hide color, and age

Characteristic	*n*	Frequency, %
Sex class (*n* = 454)		
Heifer	167	36.78
Steer	287	63.22
Breed type^1^ (*n* = 452)		
* Bos taurus*	438	96.90
* Bos indicus*	14	3.10
Hide color (*n* = 449)		
Black	286	63.70
Not black	163	36.30
Cattle age (*n* = 414)		
Under 30 mo	401	96.86
Over 30 mo	13	3.14

^1^Cattle were classified as having *Bos indicus* influence when they possessed two or more typical breed characteristics, which included having a large hump above the shoulders, droopy ears, or excess skin on the dewlap and prepuce.

### Preslaughter Management Factors

Descriptive statistics of preslaughter management factors are outlined in Table 4. In the current study, cattle were transported 68.70 ± 44.70 km (mean ± SD) and waited on the truck at the plant for an average of 54.43 ± 38.30 min. The shortest amount of time cattle waited to be unloaded was 6 min, with a maximum waiting time of 208.00 min (approximately 3.5 h). The average lairage duration was 152.07 ± 38.30 min and ranged from 92.00 to 253.00 min (approximately 4 h). Lairage density, measured in m^2^ of pen allowance per individual animal, averaged 3.67 ± 1.75 m^2^ and ranged from 2.05 to 13.24 m^2^.

**Table 4.** Description of preslaughter management factors measured as potential risk factors associated with animal welfare and meat quality outcomes

Variable^1^	*n*	Min	Mean	Max	SD
Distance traveled^2^, km	454	2.74	68.70	206.00	44.70
Truck waiting time, min	454	6.00	54.43	208.00	38.61
Lairage duration, min	446	92.00	152.07	253.00	38.30
Lairage density, m^2^/animal	451	2.05	3.67	13.24	1.75

^1^All measurements were taken on an individual animal basis.

^2^Google Maps was used to calculate an estimated transport distance between the feedlot of origin and the plant.

### Mobility

Of the 438 cattle assigned an individual mobility score, 67.12% (*n* = 294) were assigned a score of 1 (i.e., normal mobility; Table 5). Most cattle with impaired mobility were given a score of 2 (*n* = 140, 31.96%). Greater mobility scores were less frequently observed, as less than one percent of cattle included in the study scored a 3 (*n* = 4, 0.91%), and none scored a 4. The results of the mobility regression analysis identified two factors significantly associated with the outcome: lairage density and season (Table 6). A one-unit (m^2^/animal) increase of space allowance per animal in lairage was associated with decreased odds of cattle with impaired mobility (i.e., scoring a 2 or higher; OR: 0.83, CI: 0.73, 0.96; *P* = 0.011). Compared to cattle slaughtered in the summer season, those slaughtered in the winter season had reduced odds of exhibiting impaired mobility (OR: 0.26, CI: 0.13, 0.51; *P* = 0.0001). Additionally, there were statistical tendencies for truck waiting time (OR: 1.01, CI: 1.00, 1.01; *P* = 0.069), blood lactate (OR: 0.89, CI: 0.77, 1.02; *P* = 0.085), and carcass bruising (OR: 0.64, CI: 0.41, 1.00; *P* = 0.051) to be associated with impaired mobility scores.

**Table 5.** Descriptive statistics for mobility, bruise score, yield grade, and quality grade for individual animals

Variable	*n*	Frequency (%)
Mobility score (*n* = 438)		
1	294	67.12
2	140	31.96
3	4	0.91
4	0	0.00
Bruise score^1^ (*n* = 447)		
None	89	19.91
One small	72	16.11
One large	28	6.26
Multiple small	122	27.29
Multiple large	136	30.43
Yield grade (*n* = 447)		
1	37	8.28
2	255	57.05
3	139	31.10
4	15	3.36
5	1	0.22
Quality grade (*n* = 446)		
Prime	11	2.47
Choice	291	65.25
Select	137	30.72
Standard	4	0.90
Cutter	3	0.67

^1^Bruise score categories were classified as follows: none = no bruise present; one small = one bruise that was less than or equal to the size of a deck of cards; one large = one bruise that was larger than the size of a deck of cards; multiple = two or more bruises and the size of the largest bruise was noted.

**Table 6.** Multivariable logistic regression models for categorical variables, including mobility and bruising^1^

Variable	Estimate	SE	*P* value	OR (95% CI)
Mobility (*n* = 406)				
Intercept	1.1435	0.5897	0.0525	3.14 (0.99, 9.97)
Bruising				
None/minimal	*Referent*	—	—	—
Greater than minimal	−0.4435	0.2276	0.0513	0.64 (0.41, 1.00)
Lairage density, m^2^/animal	−0.1820	0.0716	0.0110	0.83 (0.73, 0.96)
Lactate, mmol/L	−0.1223	0.0711	0.0853	0.89 (0.77, 1.02)
Season				
Summer	*Referent*	—	—	—
Winter	−1.3662	0.3572	0.0001	0.26 (0.13, 0.51)
Truck waiting time, min	0.0057	0.0031	0.0693	1.01 (1.00, 1.01)
Bruising (*n* = 431)				
Intercept	1.9956	1.3381	0.1359	7.36 (0.54, 104.00)
Bodyweight, kg	−0.0026	0.0018	0.1508	0.997 (0.994, 1.00)
Mobility				
Normal	*Referent*	—	—	—
Impaired	−0.3709	0.2232	0.0965	0.69 (0.45, 1.07)
Lairage duration, min	0.0063	0.0029	0.0294	1.01 (1.00, 1.01)^2^
Season				
Summer	*Referent*	—	—	—
Winter	−0.7251	0.2429	0.0028	0.48 (0.30, 0.78)
Sex				
Steer	*Referent*	—	—	—
Heifer	−0.7620	0.2854	0.0076	0.47 (0.27, 0.81)

^1^Estimates for each variable and reported *P* values are from solutions for fixed effects of the respective regression model.

^2^95% CI has been rounded and does not include 1.

### Blood Parameters

#### Cortisol

Serum cortisol averaged 21.70 ± 11.30 ng/mL (mean ± SD) and ranged from 0.08 to 77.40 ng/mL (Table 7). Overall, there was evidence of significant associations (*P ≤* 0.05) between body weight, bruising, and truck waiting time with cortisol concentrations (Table 8). Greater average cortisol concentrations were associated with heavier cattle (*P* = 0.049), cattle that waited longer in the truck at the plant (*P* = 0.038), and those with carcass bruising compared to those without carcass bruising (23.0 ± 0.89 vs. 20.3 ± 1.05 ng/mL (predicted mean ± SE); *P* = 0.015). In contrast, there was no evidence of an association between sex or lairage density on cortisol concentrations (*P* = 0.130 and *P* = 0.128, respectively).

**Table 7.** Description of physiological stress parameters, including lactate, creatine kinase, cortisol, and postmortem muscle pH

Variable	*n*	Min	Mean	Max	SD
Cortisol, ng/mL	441	0.08	21.70	77.40	11.30
Creatine kinase, U/L	450	240.00	949.00	8622.00	933.00
Lactate, mmol/L^1^	431	0.70	5.72	14.80	2.46
Muscle pH^2^	414	5.20	5.53	6.56	0.12

^1^Lactate was analyzed in whole exsanguination blood with a portable lactate meter approximately 30 min postmortem.

^2^Muscle pH was determined with a portable pH meter approximately 32 to 36 h postmortem.

**Table 8.** Multivariable linear mixed-effect regression models for continuous variables, including cortisol, creatine kinase (CK), lactate, and muscle pH^1^

Variable	Estimate	SE	*P* value
Cortisol, ng/mL (*n* = 433)			
Intercept	7.4214	6.4020	0.2472
Bodyweight, kg	0.0189	0.0096	0.0488
Bruising			
None/minimal	*Referent*	—	—
Greater than minimal	2.6904	1.0972	0.0146
Lairage density, m^2^/animal	-0.5357	0.3510	0.1283
Sex			
Steer	*Referent*	—	—
Heifer	2.7331	1.7778	0.1304
Truck waiting time, min	0.0363	0.0173	0.0380
Creatine kinase^2^, U/L (*n* = 442)			
Intercept	666	78.7	< 0.0001
Season			
Summer	*Referent*	—	—
Winter	0.8810	0.0567	0.0553
Sex			
Steer	*Referent*	—	—
Heifer	0.8800	0.0563	0.0541
Lairage duration, min	1.0000	0.0008	0.0509
Lactate, mmol/L (*n* = 409)			
Intercept	4.3262	1.0905	< 0.0001
Bodyweight, kg	0.0028	0.0014	0.0498
Mobility			
Normal	*Referent*	—	—
Impaired	-0.3611	0.1631	0.0274
Lairage duration, min	0.0052	0.0027	0.0523
Season			
Summer	*Referent*	—	—
Winter	-3.6800	0.2798	< 0.0001
Sex			
Steer	*Referent*	—	—
Heifer	0.4660	0.3045	0.1321
Truck waiting time, min	0.0063	0.0027	0.0227
Muscle pH (*n* = 401)			
Intercept	5.6470	0.0504	< 0.0001
Bodyweight, kg	-1.666 × 10^-4^	8.786 × 10^-5^	0.0594
Mobility			
Normal	*Referent*	—	—
Impaired	-0.0269	0.0122	0.0285
Season			
Summer	*Referent*	—	—
Winter	-0.0200	0.0155	0.2056

^1^Estimates for each variable and reported *P* values are from solutions for fixed effects of the respective regression model.

^2^A logarithmic transformation was performed on CK data for analysis and back-transformed for reporting purposes.

#### Creatine Kinase

Serum CK concentrations ranged from 240.00 to 8622.00 U/L with an average value of 949.00 ± 933.00 U/L (Table 7). Results indicate that greater average CK concentrations tended to be associated with heifers versus steers (*P* = 0.054), the winter versus summer season (*P* = 0.055), and in cattle that were held in lairage for longer durations (*P* = 0.051; Table 8) but were not statistically significant (*P >* 0.05).

#### Lactate

Study cattle had a mean lactate concentration of 5.72 *± *2.46 mmol/L, ranging from 0.70 to 14.80 mmol/L (Table 7). Results of lactate analysis identified multiple factors associated with the blood parameter, including body weight, truck waiting time, lairage duration, season, and mobility (Table 8). Greater average lactate concentrations were associated with heavier cattle (*P* = 0.050) and cattle that waited on the truck for more time (*P *= 0.023). Season was associated with greater lactate, as cattle that were slaughtered in the winter had significantly lower lactate levels than cattle slaughtered in the summer (3.54 ± 0.22 vs. 7.22 ± 0.17 mmol/L (predicted mean ± SE); *P* < 0.0001). In addition, mobility score was associated with lactate, as cattle with impaired mobility had lower blood lactate than cattle with normal mobility (5.20 ± 0.18 vs. 5.56 ± 0.14 mmol/L; *P* = 0.027). Finally, there was also a tendency for lactate to be elevated in cattle held in longer lairage (*P *= 0.052).

### Meat Quality Parameters

#### Bruising

Approximately one-third (*n* = 161, 36.02%) of study cattle had either no carcass bruising (*n* = 89, 19.91%) or a single small bruise (*n* = 72, 16.11%; Table 5). A smaller proportion of cattle had a single large bruise (*n* = 28, 6.26%), with most cattle having multiple bruises of varying sizes (small: *n* = 122, 27.29%; large: *n* = 136, 30.43%). Multiple factors were associated with carcass bruising, including lairage duration, season, and sex (Table 6). Odds of carcass bruising were positively associated with lairage duration (OR: 1.01, CI: 1.0007, 1.0122; *P* = 0.029). Compared with the summer season, the winter season was associated with reduced odds of cattle being bruised (OR: 0.48, CI: 0.30, 0.78; *P* = 0.003). Similarly, the odds of having a bruise were reduced in heifers compared with steers (OR: 0.47, CI: 0.27, 0.81; *P* = 0.008). There was a weak tendency for mobility to be associated with bruising (OR: 0.69, CI: 0.45, 1.07; *P* = 0.097). In contrast, there was no evidence of an association between body weight on bruise incidence (OR: 0.997, CI: 0.994, 1.00; *P* = 0.151).

#### Muscle pH

Study carcasses had a mean postmortem muscle pH of 5.53 ± 0.12 and ranged from 5.20 to 6.56 (*n* = 414; Table 7). Less than one percent of carcasses were classified as dark cutters (pH range: 6.15 to 6.56; *n* = 3, 0.72%). Mobility was identified as a factor associated with muscle pH, as pH was lower in cattle with impaired mobility compared to cattle with normal mobility (pH of 5.54 ± 0.009 vs. 5.51 ± 0.011; *P* = 0.029; Table 8). There was also a tendency for pH to decrease with every one unit increase in body weight (*P* = 0.059), although the difference was small and likely not biologically relevant. Lastly, there was no evidence that muscle pH was associated with season (*P* = 0.206).

## Discussion

A multitude of work has focused on reducing fear, stress, and discomfort in cattle being moved through the final marketing phase by improving and promoting low-stress animal handling, transportation, and management processes during this time (Edwards-Callaway and Calvo-Lorenzo, 2020). Consumers increasingly care about how their food is raised (FMI, 2022), and companies have incorporated animal welfare into all segments of their operations and established it as a core pillar of corporate social responsibility. For example, packing companies have implemented new standards for cattle transporters, requiring drivers shipping cattle to their plants to be Beef Quality Assurance Transportation (**BQAT**) certified (Cargill, 2023). Even more recently, conversations and efforts around enhancing cattle comfort at the plant by providing shade or rubber mats in lairage, for example, have become more frequent (Davis et al., 2022b). Even still, there is limited information about the effects of preslaughter factors on animal welfare and meat quality outcomes, particularly among finished beef cattle in the US. A recent scoping review investigating the impact of the preslaughter period on beef quality outcomes reported that only 10 of the 85 studies assessed were conducted in North America, with even fewer studies (*n* = 5) conducted in the US (Sullivan et al., 2022). The objective of the current study was to track individual animals throughout the slaughter process to identify preslaughter factors associated with key welfare and quality outcomes in finished beef cattle in the US.

Although data collection occurred at a single beef processing facility, the sample population was representative of cattle entering the food supply chain from conventional beef-fattening systems in the U.S. Data from the 2016 NBQA, a tool used to benchmark progress in the U.S. beef industry, indicated the majority of all sampled cattle were black-hided and steers (57.8%, Eastwood et al., 2017; 66.5%, Boykin et al., 2017, respectively), aligning with the characteristics of the cattle population sampled in this study (approximately 60% were steers and another 60% black-hided). On average, carcasses in this study weighed 388 kg, supported by findings from the 2016 NBQA, which reported an average HCW of 390 kg among 8,493 fed steer and heifer carcasses (Boykin et al., 2017). While the primary focus of this study was to investigate the impact of preslaughter factors on mobility, blood parameters, bruising, and muscle pH for individual animals, a trend that emerged from the analysis was that animal-related factors, namely body weight, and sex, were associated with a few of the measured outcomes. The current study identified body weight as a factor associated with serum cortisol concentration and blood lactate; a 1-kg increase in body weight was associated with an increase in both blood parameters. In a study conducted in finishing pigs, live weight itself had a limited impact on blood lactate; however, the effect of handling intensity on lactate was strong, as lactate concentrations increased with increasing handling intensity (Hamilton et al., 2004); this is a finding supported by results in Hagenmaier et al. (2017) with a group of finished beef cattle. The current literature suggests that other factors, such as the quality and intensity of animal handling, significantly impact an animal’s physiological response to stress and warrant consideration. Sex was an animal-related characteristic associated with carcass bruising, i.e., heifers had less bruising than steers. Other studies have identified sex as a risk factor for bruising (Mendonça et al., 2018; Sánchez et al., 2022), although the findings are highly variable, and the reasons for this are unclear. Behavioral differences between different sex classes may explain the variation in bruise prevalence (Kline et al., 2020).

The transportation of livestock to slaughter is regarded as one of the most stressful events in an animal’s life, having implications for both animal welfare and meat quality (Miranda-de la Lama, 2014; Schuetze et al., 2017). Exposure to variable and often extreme environmental conditions, loud noises and vibrations, novel handlers and animals, and extended periods of feed and water deprivation are just a few aspects of livestock transport that may lead to cattle becoming stressed, fatigued, or injured during transport (Wigham et al., 2018). With these risk factors considered, industry-accepted recommendations and guidelines (Leary et al., 2016; NAMI, 2021), regulations governing the transport of livestock (Twenty-Eight Hour Law, 1994), and good practices in animal handling and transport (e.g., BQAT training and certification), are in place to help ensure animal welfare is maintained during this critical juncture of the final marketing phase. Transportation conditions, such as distance or duration traveled, can be highly variable across different regions and production systems, even within the U.S. Interestingly, though, transport distance was not identified as a factor associated with any measured outcome in this study. This finding is surprising because the effects of transportation have been repeatedly explored and identified as a critical preslaughter factor for a suite of welfare and quality outcomes (Schwartzkopf-Genswein et al., 2012), many of which were measured in this study (e.g., mobility, bruising, and muscle pH). There are several potential reasons for transport distance not emerging as an impacting factor in the current study. For instance, cattle were transported from their feedlot of origin to the plant for relatively short distances, as the mean transport distance was 68.7 km, and no cattle traveled greater than 206.0 km. For reference, the 2016 and 2022 NBQA data for fed heifers and steers indicates that mean values for distance traveled were 218.5 km and 245.3 km, respectively (Eastwood et al., 2017; NBQA., 2022). Additionally, many other transport-related characteristics, including driver experience (Tarrant, 1990), trailer type (Silva et al., 2016), loading density (Tarrant et al., 1992; Bethancourt-Garcia et al., 2019), and trailer microclimate (Schuetze et al., 2017), have been shown to impact beef cattle welfare and meat quality. Although these factors were not assessed in this study, it is possible that they may explain the variation in the measured outcomes better than transport distance alone and should be considered in future work.

Once cattle arrive at the slaughter plant they often must wait to be unloaded for various reasons (e.g., pen capacity, plant breaks, arrivals outside of the scheduled time; Davis et al., 2024). To the author’s knowledge, the impact that the time cattle wait to unload upon arrival to the plant (i.e., truck waiting time) has on cattle welfare and meat quality has not been studied. However, truck wait time is a component of the NAMI Transportation Audit (NAMI, 2021). The audit criterion indicates that trucks should be unloaded within 60 min of arrival time to the plant; each additional 30 min of waiting time results in a one-point deduction on the audit, and trucks that wait for longer than 120 min without reason receive zero points (NAMI, 2021). It is unclear within the audit guidelines why 60 min is considered an acceptable waiting time since this threshold’s acceptability has not been previously explored. On average, study cattle waited to unload for approximately 54 min, under the 60-min threshold determined by NAMI. However, it should also be noted that there was a small subset of study cattle that waited for extended periods, i.e., 32 of 454 cattle waited longer than 120 min, of which twelve waited between 3 and 3.5 h to unload in the summer season (which is almost two and a half times longer than the audit criteria indicates). Processing plants make concerted efforts to minimize truck waiting times by optimizing purchasing and scheduling logistics. However, a combination of internal (e.g., facility limitations, unexpected operating delays, shift changes) and external (e.g., feedlot logistics, early or late arrivals, road conditions) factors make managing truck wait times at the plant a challenge. Still, the long wait times recorded in this study, even though infrequent, are significant and warrant attention. More research exploring the effects of truck wait time on welfare and meat quality outcomes is needed to determine if the currently accepted guidelines for wait time to unload are acceptable or need revision.

In the current study, truck waiting time was identified as a factor associated with two of the three blood parameters indicative of stress (cortisol and lactate), with increased cortisol and lactate concentrations observed with increasing truck waiting times. While other studies have not directly evaluated the impacts of truck waiting time, research has assessed physiological reactions to stress in relation to being transported. Romero et al. (2014) and Capra et al. (2019) measured cortisol on the farm before transport and at the time of slaughter and observed significant increases in cortisol in cattle transported for more prolonged periods. In addition, Gruber and others (2010) measured behavioral and physiological reactions to stress after a 64 km transport to a commercial slaughter facility. Cattle that exhibited behavioral symptoms of stress post-transport had greater plasma lactate concentrations at slaughter, potentially indicative of an adrenergic stress response to transport itself (Gruber et al., 2010). Furthermore, even though the impact of the time cattle wait to unload at the plant has not been quantified, a truck’s microclimate (e.g., heat, humidity, noxious gas concentration, and overall air quality) at rest is different than when in motion (Goldhawk et al., 2014; Scheuetze et al., 2017); in particular, ventilation and airflow are altered which could have deleterious effects on cattle. Seventeen of the 32 cattle that waited for longer than 120 min on the truck were slaughtered in the summer season. The average temperature during this season was 33.9 °C (range: 26.7 to 42.2 °C) with an average Temperature Humidity Index (**THI**) of 78.2 (range: 73.0 to 83.8). According to Meat and Livestock Australia, the mean THI observed in this study (78.2) is considered Mild Stress, and the maximum THI (83.8) is considered Severe Stress (LiveCorp and Meat and Livestock Australia Veterinary Handbook, 2023). Although unknown, the effects of longer wait times coupled with high temperatures and heat load risk (i.e., Mild/Severe Stress) could have significant implications for animal welfare and meat quality and should be explored further. In market-weight pigs, an exploration of the effects of additive stressors (i.e., handling intensity, floor space during transport, and distance handled) on a host of metabolic responses found that blood lactate concentration increased linearly as pigs experienced more stressors throughout the preslaughter period (Ritter et al., 2009). Therefore, the time cattle wait to unload likely has cumulative effects on an animal’s physiological reaction to stress, and it is just one of many factors that may negatively impact an animal in the preslaughter phase.

Although regulatory requirements governing the humane slaughter of livestock do not explicitly stipulate how much space an animal should be given in holding pens at the slaughter facility, federal regulations do require that animals have access to water in pens, are provided feed if held for longer than 24 h and are given adequate space to lie down if held overnight (USDA FSIS, 1987). Additionally, industry guidelines, such as those developed by NAMI, provide guidance on how much space cattle should be allotted in holding pens and indicate that space allocations could vary based on weather, animal size, and duration of lairage (NAMI, 2021). In brief, NAMI recommendations indicate that a 545-kg animal should be allotted 1.87 m^2^ in lairage, which increases with heavier body weights (up to 2.22 m^2^; NAMI, 2021); the creation and implementation of a tiered, weight-based pen density system was added to the NAMI guidelines in a 2017 revision (Kline et al., 2019). Cattle in the current study weighed 610 kg on average and were allotted an average of 3.67 m^2^ of lairage pen space each, which is more space than the largest allotment recommended by NAMI. Even still, pen density was associated with an important welfare indicator; cattle with less space in lairage were at greater risk for having impaired mobility, potentially indicating that the currently accepted recommendations for lairage density may need to be reevaluated as cattle characteristics continue to evolve (i.e., type, body weight, frame size).

A limited number of studies have assessed the effects of pen density on welfare (Davis et al., 2022a) or meat quality outcomes in beef cattle (Sullivan et al., 2022). Also, to the authors’ knowledge, studies have yet to be conducted on the impact of lairage density on beef cattle mobility. Mobility represents a significant challenge for the beef industry as it has implications for animal welfare and production efficiency (Edwards-Callaway et al., 2017). Mobility challenges at the plant can reduce operating speeds, which can have a significant economic cost vialost production minutes. A decade ago, highly publicized reports of cattle arriving at slaughter plants with severely impaired mobility heightened industry stakeholder’s interest in and concern for cattle mobility (Beef Magazine, 2013; NPR, 2013). Since then, a tremendous amount of effort has focused on understanding risk factors associated with impaired mobility to assess and manage it more effectively (Boyd et al., 2015; Hagenmaier et al., 2017; Lee et al., 2018; Mijares et al., 2021). Even still, challenges persist in assessing and managing cattle mobility because of numerous preslaughter, animal-level, and management-related risk factors, including but not limited to environmental conditions (González et al., 2012), lairage duration (Hagenmaier et al., 2017), body weight (Edwards-Callaway et al., 2017), sex (Lee et al., 2018), and days on feed (**DOF**, Mijares et al., 2021). Although pen density has not yet been identified as a risk factor for impaired mobility, future research should explore how cattle spend their time in lairage. Since overcrowding cattle in lairage pens negatively impacts their ability to comfortably lie down or move around freely, this could worsen mobility outcomes and is an area worthy of consideration.

Cattle in high-density pens are more susceptible to heat stress because their ability to dissipate a high heat load effectively is impaired (Edwards-Callaway et al., 2021); increases in stocking densities can impact a pen’s microclimate by reducing overall airflow and increasing humidity (Weeks, 2008; Davis et al., 2022b). It should be noted that observers in the current study assessed cattle for signs of heat stress (i.e., open-mouth breathing) during lairage using a modified version of a panting score system developed by Mader et al. (2006), but due to no heat stress signs observed, it was not analyzed. Many challenges are associated with observing heat stress in lairage pens; numerous factors, such as the overcrowding of cattle in pens, time of observation, and severity of heat stress, all impact a researcher’s ability to observe it. In recent years, particularly in light of extreme heat events and cattle death loss in feedlots (Drovers, 2022; NPR, 2022), a heightened focus has been placed on the importance of providing cattle with heat mitigation, such as sprinklers, fans, shade, or a combination thereof during lairage (Davis et al., 2022b). In the current study, sprinklers were used in lairage as a form of heat mitigation when temperatures exceeded 26.7 °C; however, depending on where cattle were standing in the pen and the consistency of the sprinkler application, cattle may not have had equal access to this form of heat mitigation. Future research should explore the application and effectiveness of shade provision in lairage and the impact of pen-level temperature and humidity on animal-based outcomes.

The findings of this study indicate that seasonality was a significant risk factor for mobility and bruising, as the summer season was associated with a higher risk for impaired mobility and the incidence of ‘Greater than Minimal’ carcass bruising. In a study by Mijares et al. (2021), a greater THI was associated with an increase in the percentage of cattle scoring a two or greater on the NAMI mobility scoring system; in their study (holding all other factors constant), an increase on the THI scale from Mild Stress (i.e., 72 ≤ THI < 79) to Severe Stress (i.e., 79 ≤ THI < 89) increased the relative risk of mobility scores of two or greater by approximately 125%. In addition, although season has repeatedly been identified as a risk factor for carcass bruising (Huertas et al., 2015), the findings are highly variable across the available literature and contribute to the ongoing challenges associated with assessing and managing bruising in the beef supply chain. Comparing risk factors associated with bruising across studies is difficult because numerous variables, including sex class, animal type, quality of animal handling and facilities, and methodologies used to quantify and characterize bruising, may confound the prevalence of bruising (Sullivan et al., 2022). Although animal handling was not assessed in the current study, we can postulate that mobility and bruising were potentially related. For example, if an animal was more challenging to move (e.g., it had impaired mobility), that is potentially when handling outcomes worsened. In a population of culled cattle, Strappini et al. (2013) tracked the incidence of traumatic events in the preslaughter period and their relationships to carcass bruising; they discovered that 27% of bruises resulted from negative human–animal interactions, which occurred most frequently during the loading and unloading of animals (Strappini et al., 2013). This research emphasizes the importance of proper animal handling at the plant and identifies an area in need of additional exploration in the future.

Many factors, including lairage duration, lairage density, environmental conditions, animal condition, and others, interact with each other to have multifactorial effects on bruising, underscoring the complexity associated with pinpointing factors influencing the quality defect. There is an animal welfare component to bruising, and bruising has numerous downstream effects estimated to cost the U.S. beef industry millions of dollars annually (Lee et al., 2017). This loss is attributed to overall reductions in carcass yield due to trimming, the devaluing of cuts affected by bruising, and the increased labor needed to trim bruises (McNally and Warriss, 1996). Therefore, substantial work has been done to understand the risk factors associated with bruising to reduce its prevalence (Kline et al., 2020; Sánchez et al., 2022; Zanardi et al., 2022). In the current study, the risk of carcass bruising was positively associated with lairage duration and is consistent with other work that found that bruising worsens with longer durations of lairage (Warriss, 1990; Hoffman and Lühl, 2012). As cattle spend more time in the lairage pens, they have more opportunities to become bruised through negative animal–animal, human–animal, and animal-facility interactions (Strappini et al., 2013). Strappini et al. (2013) found that approximately one-third of bruising in their study was inflicted during the lairage period. Some extent of pain, suffering, and distress is associated with a forceful impact that would cause a bruise on an animal (Edwards-Callaway and Kline, 2020), a notion supported by findings of this study; greater average cortisol concentrations were associated with cattle that had carcass bruising compared to those without bruising, indicating some level of distress was experienced by the impacted animal. However, it should be noted that the observed differences in cortisol concentrations in the current study were minor and may be biologically irrelevant. Still, Warriss and Brown (1985) measured cortisol in exsanguination blood and found a positive correlation between plasma cortisol concentration and carcass bruising in pigs, demonstrating that a relationship between carcass bruising and plasma cortisol may truly exist.

A large body of work exists on the impact of preslaughter factors on postmortem muscle pH in beef cattle. A scoping review about the effect of preslaughter management practices on meat quality outcomes identified that pH was the most highly researched outcome among papers included in the review (i.e., pH was assessed in nearly 70% of the included studies; Sullivan et al., 2022). The mean pH of carcasses measured in this study was 5.53 and fell within the acceptable range of ultimate pH for beef (5.5–5.6; Sakowski et al., 2022). Interestingly, less than 1% of all study carcasses (3 of 414) exhibited some degree of dark cutting. Other extensive, epidemiolocal-type studies have also evaluated dark cutting in beef cattle and found similarly low incidence rates (0.3% to 1.6% among 724,639 cattle, Kreikemeier et al., 1998; 0.05% to 0.64% among 2.6 million cattle, Scanga et al., 1998; and 2.8% among 142,228 cattle, Steel et al., 2021). Due to a low incidence of dark cutters in the sample population, exploring the impact of the preslaughter management factors measured in this study on dark cutting was not possible.

In an exploration of dark cutting in a population of nearly 150,000 Australian grain-fed beef carcasses, Steel and others (2021) reported that environmental conditions (e.g., increased wind speeds and rain during lairage) and lairage duration were associated with an increased risk of dark cutting, a finding supported by a large body of work. Warren et al. (2010) found that cattle lairaged overnight were more likely to dark cut than cattle slaughtered on the day of their arrival, and Loredo-Osti et al. (2019) reported that a reduction in lairage time from 14.9 to 3.0 h would decrease the probability of dark cutting from 7.21% to 0.02%. Other studies have documented numerous other factors associated with dark cutting. For example, time of the year (Kreikemeier et al., 1998; McGilchrist et al., 2014); duration of transport (Gallo et al., 2003; Chulayo and Muchenje, 2017; Burns et al., 2019), and animal-related factors (Scanga et al., 1998) are just a few that have been reported previously. Identifying a single preslaughter factor associated with this defect is challenging, mainly because numerous and compounding intrinsic and extrinsic factors contribute to its occurrence. While dark cutting is a costly quality defect (carcasses can be discounted between 20 and 55 dollars per cwt, USDA AMS., 2024) and there is an economic incentive to minimize it, it is extremely difficult to manage; factors contributing to its occurrence exist throughout the entire final marketing phase, some of which cannot be controlled (e.g., fluctuations in temperature 24 h prior to slaughter; Scanga et al., 1998). To thoroughly investigate relationships between preslaughter factors and dark cutting, future research may explore dark cutting in targeted cattle populations that may be at higher risk for dark cutting or during specific times of the year when dark-cutting incidence is known to be higher.

Preslaughter factors (e.g., truck waiting time and lairage duration) differ by production shift, with many large commercial beef processing plants in the U.S. operating on two 8-h shifts. The plant may experience unexpected downtime, delays in receiving cattle, or pen capacity limitations throughout the day, which could potentially exasperate some of the factors measured in this study. One limitation of the current study is that samples were collected only from cattle slaughtered on the first shift. Across two shifts, the trends are likely similar to those found in the current study. However, the magnitude of these relationships might differ if animals arriving during the second shift were included in this study. Although animal behavior and handling outcomes were not assessed in the present study, variations in behavior and handling are likely associated with many of the studied outcomes—namely, mobility and bruising. Future research should investigate how cattle spend their time in lairage and, more specifically, if their access to water, space to lie comfortably, and room to move around freely impacts animal-based outcomes. Further, the relationship between mobility and handling outcomes and its downstream effects on bruising and muscle pH could lead to understanding how best to manage cattle with impaired mobility in the preslaughter phase to maximize welfare and quality outcomes.

The results of this study support a growing body of work highlighting the importance of the preslaughter period for animal welfare and meat quality. This project also identifies areas for continuous improvement, such as reducing the time trucks wait to unload at the plant, shortening the duration of lairage, and providing cattle with more space in lairage pens. Additionally, there was a seasonal effect on many of the measured outcomes. Although environmental conditions (e.g., temperature, humidity, wind speeds) are challenging to manage, there are opportunities for plants to modify their practices at times of the year when cattle are most susceptible to adverse welfare outcomes. Providing heat mitigation (i.e., shade and more efficient sprinkler systems) during lairage may help cattle cope with high heat loads and is just one example of the opportunities that exist to improve the welfare of finished cattle arriving at slaughter plants. However, additional exploration is needed to quantify the effects of shade on cattle at plants. Presumably, exposure to many stressors during the final marketing phase has a compounding effect on cattle welfare and meat quality, and the factors identified in this study are just a few of many important preslaughter factors requiring further investigation. Lastly, a significant amount of work has been done to improve the transport, handling, and management of cattle in the preslaughter phase, which is evident in the findings of this study. Continuing to promote and improve good practices in animal handling and transport will be key to advancing animal welfare in the beef supply chain. Looking ahead, it will become more important to understand the economic impacts of adverse welfare outcomes for processors and the role that corporate sustainability goals play in promoting animal welfare.
